# Role in staging and prognostic value of pretherapeutic F-18 FDG PET/CT in patients with gastric MALT lymphoma without high-grade transformation

**DOI:** 10.1038/s41598-021-88815-2

**Published:** 2021-04-29

**Authors:** Yong-Jin Park, Seung Hyup Hyun, Seung Hwan Moon, Kyung-Han Lee, Byung Hoon Min, Jun Haeng Lee, Won Seog Kim, Seok Jin Kim, Joon Young Choi

**Affiliations:** 1 Department of Nuclear Medicine, Samsung Medical Center, Sungkyunkwan University School of Medicine, 81, Irwon-ro, Gangnam-gu, Seoul, 06351 Republic of Korea; 2 Division of Gastroenterology, Department of Internal Medicine, Samsung Medical Center, Sungkyunkwan University School of Medicine, Seoul, 06351 Republic of Korea; 3 Division of Hematology-Oncology, Department of Internal Medicine, Samsung Medical Center, Sungkyunkwan University School of Medicine, Seoul, 06351 Republic of Korea; 4 Department of Nuclear Medicine, Soonchunhyang University Cheonan Hospital, 31, Suncheonhyang 6-gil, Dongnam-gu, Cheonan, Chungcheongnam-do 31151 Republic of Korea

**Keywords:** Cancer imaging, Gastrointestinal cancer, Haematological cancer

## Abstract

The purpose of this retrospective study was to investigate the role in staging and prognostic value of pretherapeutic fluorine-18-fluorodeoxyglucose (F-18 FDG) positron emission tomography (PET)/computed tomography (CT) in patients with gastric mucosa-associated lymphoid tissue (MALT) lymphoma without high-grade transformation (HT). We retrospectively reviewed 115 consecutive patients with histopathologically confirmed gastric MALT lymphoma without HT who underwent pretherapeutic F-18 FDG PET/CT. Kaplan–Meier and Cox proportional-hazards regression analyses were used to identify independent prognostic factors for disease free survival (DFS) among 13 clinical parameters and three PET parameters. In two of 115 patients (1.7%), the clinical stage appeared higher according to F-18 FDG PET/CT. In univariate analysis, *Helicobacter pylori* (HP) infection (P = 0.023), treatment modality (P < 0.001), and stage including PET/CT (P = 0.015) were significant prognostic factors for DFS. In multivariate analysis, only treatment modality was an independent prognostic factor (P = 0.003). In conclusion, F-18 FDG PET/CT played an important role in enabling upstaging of patients with gastric MALT lymphoma without HT. F-18 FDG PET/CT may have a prognostic role in gastric MALT lymphoma without HT by contributing to better staging.

## Introduction

Marginal zone B cell lymphoma (MZL) is the second most common type of indolent B cell non-Hodgkin’s lymphoma (NHL) and accounts for about 6% of all lymphoid neoplasms^1^. MZL is derived from post-germinal center B cells in the marginal zone of mucosa-associated lymphoid tissue (MALT), and consists of three types: extranodal, nodal, and splenic MZL^2,3^. Extranodal, nodal, and splenic MZL accounted for 70%, 20%, and 10% of all MZL cases based on 2016 World Health Organization data^1^. Extranodal MZL is also referred to as MALT lymphoma and defined as lymphoma contained MALT that can damage any mucosal site^4^. Gastric MALT lymphoma accounts for about 35% of all primary gastric lymphomas, and the stomach is the most common site of MALT lymphoma^5,6^. Gastric MALT lymphoma is strongly associated with *Helicobacter pylori* (HP) infection^7^.

Primary gastric lymphoma, including gastric MALT lymphoma and gastric diffuse large B-cell lymphoma (DLBCL), is the most common form of extranodal NHL^8^. High-grade transformation (HT) is one of the ways in which a gastric DLBCL is developed from gastric MALT lymphoma, some gastric DLBCL may develop from indolent gastric MALT lymphoma via step of HP-dependent HT and residual gastric DLBCL may develop along antigen-independent pathways as de novo gastric DLBCL^8^. When gastric MALT lymphoma and gastric DLBCL were simultaneously identified in the initial workup, it was determined that there was the HT of gastric MALT lymphoma. In general, this hypothesis seems reasonable, as HT is thought to result from HP-dependent gastric MALT lymphoma. HT and gastric DLBCL share numerous biological characteristics^8^. In a previous study, gastric MALT lymphoma without HT had a better prognosis than gastric MALT lymphoma with HT. A 10-year survival rate for gastric MALT lymphoma is near to 90% with a disease-free survival (DFS) of approximately 70%. However, gastric MALT lymphoma can progress and transform into DLBCL whereby the 10-year survival rate drops to about 42%^9^. In a previous study of primary gastric lymphoma, maximum standardized uptake values (SUV_max_) of gastric DLBCL had a higher SUV_max_ and a worse prognosis for the overall survival than gastric MALT lymphoma^6^. Therefore, it elucidated that gastric MALT lymphoma without HT had a lower fluorine-18-fluorodeoxyglucose (F-18 FDG) avidity than that of gastric MALT lymphoma with HT. Only 7.5% of marginal zone lymphoma became DLBCL by HT, and a high percentage of gastric MALT lymphoma was not accompanied by HT. In addition, HT did not appear in early pathogenesis of gastric MALT lymphoma^10^. For investigating an indolent gastric MALT lymphoma without HT, study of the clinical role of F-18 FDG positron emission tomography (PET)/computed tomography (CT) in gastric MALT lymphoma without HT is needed.

The initial staging workup includes esophagogastroduodenoscopy with multiple biopsies, endoscopic ultrasound, CT scans, physical examination, and laboratory parameters for suspected patients with gastric MALT lymphoma^11,12^. F-18 FDG PET/CT is controversial and lacks evidence of clinical usefulness in the initial staging workup^11^. The modified Ann Arbor staging system, Lugano system, and Paris staging system are used to determine clinical staging of gastric MALT lymphoma^11,12^. F-18 FDG PET/CT is a keystone of staging in Hodgkin’s lymphoma (HL) and aggressive NHL^13^, but the clinical role of F-18 FDG PET/CT in gastric MALT lymphoma is not yet obvious^4,14^. In previous studies, a small number of patients with gastric MALT lymphoma were upstaged by F-18 FDG PET/CT^4,14^.

Predicting the prognosis of F-18 FDG PET/CT in lymphoma can help clinicians to provide patient tailored therapy, reduce toxicity, and improve patient outcomes^15,16^. In addition, prediction of prognosis is valuable because it can be used to change treatment plans and prevent unnecessary treatment^14^. Several prognostic factors for MALT lymphoma and gastric MALT lymphoma are known from previous studies. The MALT lymphoma International Prognostic Index (MALT-IPI) classified low, intermediate, and high-risk patients using three individual features including age over 70 years, Ann Arbor stage III or IV, and elevated lactate dehydrogenase (LDH)^17^. In previous studies of gastric MALT lymphoma, high IPI score, elevated erythrocyte sedimentation rate, and low platelets are known as prognostic factors for overall survival, and depth of remission is a prognostic factor for relapse free survival^18,19^. F-18 FDG PET/CT plays an important role in the prognosis of lymphoma^13^, but there remains controversy about the prognostic role of F-18 FDG PET/CT in patients with MALT lymphoma^5^.

There have been previous studies on the role in staging and prognostic value of F-18 FDG PET/CT in patients with primary gastric lymphoma^6,20,21^. However, there were no previous studies dealing with the role in staging of F-18 FDG PET/CT and prognostic value of F-18 FDG PET/CT for DFS in gastric MALT lymphoma without HT at the time of initial diagnosis. Therefore, we investigated the role in staging and prognostic value of pretherapeutic F-18 FDG PET/CT in patients with gastric MALT lymphoma without HT.

## Materials and methods

### Study population

This retrospective study reviewed the electronic medical records of patients with gastric MALT lymphoma without HT who underwent pretherapeutic F-18 FDG PET/CT for initial staging workup at our medical center. Inclusion criteria were as follows (n = 170): (1) consecutive patients with histopathologically confirmed gastric MALT lymphoma from August 2008 to December 2014, and (2) patients who underwent pretherapeutic F-18 FDG PET/CT at our medical center. Exclusion criteria were as follows (n = 55): (1) patients who underwent pretherapeutic F-18 FDG PET/CT at another medical center (n = 6), (2) patients with different types of cancers (n = 28) and pathologically confirmed gastric DLBCL (n = 15), and (3) patients who were lost to follow-up immediately after diagnosis (n = 6). When gastric MALT lymphoma and gastric DLBCL were simultaneously identified by multiple endoscopic biopsies in the initial staging workup, it was determined that there was the HT of gastric MALT lymphoma. The 28 patients with different types of cancers were five stomach cancer, four cholangiocarcinoma, four lung cancer, three thyroid cancer, two colon cancer, two rectal cancer, one stomach and colon cancers, one hepatocellular carcinoma, one breast cancer, one uterine cervical cancer, one uterine endometrial cancer, one ovarian cancer, one renal cell carcinoma, one acute myeloid leukemia, and one mantle cell lymphoma. Finally, a total of 115 patients (53 men and 62 women; age range of 23 to 77 years) were enrolled in this study. We monitored recurrences of gastric MALT lymphoma from August 2008 to June 2017. The mean follow-up period (SD) was 42 (21) months (range 0 to 95 months). The Institutional Review Board of Samsung Medical Center approved this retrospective study (2020-03-201-001), and waived the requirement for informed consent. All procedures performed in the study involving human participants were in accordance with the ethical standards of our institutional review board and with the 1964 Helsinki declaration and its later amendments or comparable ethical standards.

### Staging, response evaluation, recurrence evaluation, and follow-up

In this study, patients were staged according to the modified Ann Arbor staging system. Lesions confined to the stomach were classified as stage IE, and lesions involving the stomach and the perigastric lymph nodes (IIE1) or sub-diaphragmatic lymph nodes (IIE2) were classified as stage IIE. Stage IIIE includes patients with involvement of the gastrointestinal tract or/and lymph nodes on both sides of the diaphragm. Stage IVE includes patients with extension to the extra-gastrointestinal tissue or organs^22^.

The Groupe d’ Etude des Lymphomes de l’ Adulte (GELA) histopathological grading system of post-treatment gastric biopsies was based on histopathological findings. This response system consists of complete response (CR), probable minimal residual disease (pMRD), responding residual disease (rRD), and no change (NC)^18^. In this study, pMRD and pRD were considered partial response (PR), and NC was considered stable disease (SD). In cases of radiologically measurable disease, treatment response was classified as CR, PR, SD, or progressive disease (PD) by RECIST 1.1 and PERCIST 1.0^23^. We defined clinical CR for patients with both histopathological CR and radiologic CR in this study.

In this study, 12 (10.4%) patients who had clinical CR after treatment exhibited recurrence in regular follow-up. The regular follow-up examinations included esophagogastroduodenoscopy with multiple biopsies and/or systemic staging modalities including CT scans of thorax and abdomen and/or F-18 FDG PET/CT. In general, regular follow-up was performed every 3 months during the first 2 years and every 6 or 12 months thereafter.

### F-18 FDG PET/CT imaging

All patients fasted for at least 6 h before F-18 FDG PET/CT. Blood glucose level was measured before F-18 FDG injection, and the injection was given when the level was less than 200 mg/dL. No intravenous or oral contrast medium was used during F-18 FDG PET/CT scans. At our medical center we performed F-18 FDG PET/CT scans using two different PET/CT scanners (Discovery LS and Discovery STE, GE Healthcare, Milwaukee, WI, USA). Of the 115 patients, 34 were examined using a Discovery LS PET/CT scanner and 81 were examined using a Discovery STE PET/CT scanner. In the Discovery LS PET/CT scanner, whole-body CT was performed by a continuous spiral method using an eight-slice helical CT 60 min after injection with 5.0 MBq/kg (135.14 μCi/kg) of F-18 FDG. CT images were obtained with acquisition parameters of 140 keV, 40–120 mAs adjusted to the patients’ body weight and a section width of 5 mm. After the CT scan, emission scans were obtained from the base of the skull to the thigh for 4 min/frame in two-dimensional (2-D) mode. Attenuation-corrected PET images were reconstructed using CT images and a 2-D ordered subsets expectation maximization algorithm with two iterations and 28 subsets. Voxel size was 4.3 × 4.3 × 3.9 mm. In the Discovery STE PET/CT scanner, whole-body CT was performed by a continuous spiral method using a 16-slice helical CT 60 min after the injection with 5.0 MBq/kg (135.14 μCi/kg) of F-18 FDG. CT images were obtained with acquisition parameters of 140 keV, 30–170 mAs with AutomA mode and a section width of 3.75 mm. After the CT scan, emission scans were obtained from the base of the skull to the thigh for 2.5 min/frame in three-dimensional (3-D) mode. Attenuation-corrected PET images were reconstructed using CT images and a 3-D ordered subsets expectation maximization algorithm with two iterations and 20 subsets. Voxel size was 3.9 × 3.9 × 3.3 mm. Standardized uptake value (SUV) was calculated by adjusting for the injected dose of F-18 FDG and patient body weight. Advantage Workstation VolumeShare 7 (AW 4.7, GE Healthcare) was used to perform PET/CT image co-registration.

### Image analysis of F-18 FDG PET/CT

Two nuclear medicine physicians with more than 5 years of experience interpreted F-18 FDG PET/CT through mutual agreement and used Advantage Workstation VolumeShare 7 (AW 4.7, GE Healthcare). These physicians conducted visual analyses of F-18 FDG PET/CT with knowledge of the stomach involved by histopathologically confirmed gastric MALT lymphoma. If F-18 FDG uptake in the histopathologically confirmed location of gastric MALT lymphoma was higher than the physiologic F-18 FDG uptake of nearby normal tissue, or F-18 FDG uptake was shown in abnormal wall thickening on CT images of PET/CT scans, it was considered focal uptake. In contrast, it was considered diffuse uptake if F-18 FDG uptake in the histopathologically confirmed location of gastric MALT lymphoma was not distinct from physiologic F-18 FDG uptake of nearby normal tissue or diffuse F-18 FDG uptake was shown along the gastric wall without wall thickening. For staging, we identified additional lymphoma involvement if focal F-18 FDG uptake was observed where normal physiologic F-18 FDG uptake was not shown. However, lymph nodes accompanied by fatty hilum, high attenuation, or calcification on CT images were considered reactive lymph nodes rather than indicators of lymphoma involvement even if we observed focal F-18 FDG uptake^24,25^. For semi-quantitative analysis of gastric MALT lymphoma SUV_max_ was obtained at pathologically confirmed locations of gastric MALT lymphoma regardless of the F-18 FDG avidity of gastric MALT lymphoma, and mean standardized uptake value (SUV_mean_) of normal gastric background was obtained at pathologically unconfirmed locations of gastric MALT lymphoma. SUV harmonization of different PET/CT scanners was not performed.

### Clinical parameters and PET parameters

A total of 16 parameters were investigated to identify independent prognostic factors for DFS. Of the 16 parameters, 13 were clinical parameters and three were PET parameters. The clinical parameters included age, sex, β-2 microglobulin, hemoglobin, LDH, bone marrow involvement, B symptom, International Prognostic Index (IPI) score, extragastric spread, HP infection, treatment modality, stage including F-18 FDG PET/CT, and stage excluding F-18 FDG PET/CT. Treatment modality is defined as consisting of only HP eradication and other treatments. The PET parameters included SUV_max_ of gastric MALT lymphoma, tumor-to-normal tissue (T/N) ratio, which was defined as SUV_max_ of gastric MALT lymphoma divided by SUV_mean_ of normal gastric background, of gastric MALT lymphoma, and F-18 FDG uptake pattern of gastric lesions. Information about each parameter is summarized in Table 1.

**Table 1:** Baseline characteristics of 115 patients.

Parameter	N (proportion)	Mean ± SD (range)
Age (years)		53 ± 11 (23–77)
**Sex**		
Men	53 (46.1)	
Women	62 (53.9)	
Recurrence	12 (10.4)	
DFS (months)		42 ± 21 (0–95)
β-2 microglobulin (mg/L)		1.68 ± 0.95 (0.54–7.37)
Hemoglobin (g/dL)		13.5 ± 2.0 (6.5–17.7)
LDH (IU/L)		357 ± 77 (232–702)
Bone marrow involvement	4 (3.5)	
B symptom	5 (4.3)	
**IPI score**		
0	72 (62.6)	
1	35 (30.4)	
2	8 (7.0)	
Extragastric spread	15 (13.0)	
HP infection	96 (83.5)	
**Treatment modality**		
Only HP eradication	87 (75.7)	
Other treatments	28 (24.3)	
**Stage including F-18 FDG PET/CT**		
I	103 (89.6)	
II	4 (3.5)	
III	1 (0.9)	
IV	7 (6.1)	
**Stage excluding F-18 FDG PET/CT**		
I	105 (91.3)	
II	3 (2.6)	
III	0 (0)	
IV	7 (6.1)	
SUV_max_ of gastric MALT lymphoma		4.35 ± 1.76 (1.45–10.24)
T/N ratio of gastric MALT lymphoma		2.00 ± 0.80 (0.89–6.51)
**F-18 FDG uptake pattern of gastric lesion**		
Diffuse	67 (58.3)	
Focal	48 (41.7)	

SD, standard deviation; DFS, disease free survival; LDH, lactate dehydrogenase; IPI, international prognostic index; HP, *helicobacter pylori*; F-18 FDG, fluorine-18-fluorodeoxyglucose; PET, positron emission tomography; CT, computed tomography; SUV_max_, maximum standardized uptake value; MALT, mucosa-associated lymphoid tissue; T/N ratio, tumor-to-normal tissue ratio.

### Statistical analysis

Dichotomous parameters were used for statistical analysis in the present study. The optimal cutoff values of SUV_max_ and T/N ratio of gastric MALT lymphoma were calculated by time-dependent receiver operating characteristic (ROC) curve analysis. It was performed using R version 4.0.0 (R Core Team (2020). R: A language and environment for statistical computing. R Foundation for Statistical Computing, Vienna, Austria. URL http://www.R-project.org/) and survivalROC package version 1.0.3 (Patrick & Paramita, 2013).

Because no patients included in the present study died, we defined an event as recurrence and performed univariate and multivariate analyses for DFS. DFS was defined a period during which there was no evidence of recurrence or death after successful treatment. The statistical analysis was performed using MedCalc statistical software version 12.7.5.0 for Windows (Ostend, Belgium). We used the Kaplan–Meier method with a log-rank test for univariate analysis. We performed a multivariate analysis using parameters with P values less than 0.05 in the univariate analysis. If the P value was less than 0.05, we rejected the null hypothesis, which was a general statement of no difference. Cox proportional-hazards regression with enter method was used in multivariate analysis. In multivariate analysis, parameters with P values less than 0.05 were used to identify independent prognostic factors for DFS. In addition, multiple variable graphs were used to compare SUV_mean_s of normal gastric background, SUV_max_s of gastric MALT lymphoma, and T/N ratios of gastric MALT lymphoma between all patients, patients who underwent Discovery STE, and patients who underwent Discovery LS. An independent T-test was used to compare the mean values of SUV_mean_s of normal gastric background measured by the two different PET/CT scanners.

### Ethical declarations

All procedures performed in the study involving human participants were in accordance with the ethical standards of our institutional review board and with the 1964 Helsinki declaration and its later amendments or comparable ethical standards.

### Consent to participate

The Institutional Review Board of Samsung Medical Center approved this retrospective study (2020-03-201-001), and the requirement to obtain informed consent was waived.

## Results

### Patient characteristics

The characteristics of the 115 enrolled patients are summarized in Table 1. Of these patients, 12 patients (10.4%) experienced recurrence, but none died during follow-up. Ninety-six (83.5%) were confirmed to have HP infections, while the remaining 19 (16.5%) were not confirmed to have HP infections. Eighty-seven (75.7%) were treated with only HP eradication, while the remaining 28 (24.3%) were treated with other treatments. Most of the study patients were in modified Ann Arbor stage I. One hundred three (89.6%) were in modified Ann Arbor stage I during the initial staging workup including F-18 FDG PET/CT, and 105 (91.3%) were modified Ann Arbor stage I during the initial staging workup excluding F-18 FDG PET/CT. Mean SUV_max_ of gastric MALT lymphoma was 4.35, and the SUV_max_ was measured with varying F-18 FDG uptakes from 1.45 to 10.24. The mean T/N ratio of gastric MALT lymphoma was 2.00. In cases of F-18 FDG uptake pattern of gastric lesion, 48 (41.7%) were observed as having focal uptake, while the remaining 67 (58.3%) were observed as having diffuse uptake.

### Staging role of F-18 FDG PET/CT

In two of 115 patients (1.7%), clinical stage was upstaged by pretherapeutic F-18 FDG PET/CT. In the first case, a 71-year-old female patient was initially diagnosed with modified Ann Arbor stage IE and then upstaged to IIIE by F-18 FDG PET/CT. A hypermetabolic paraesophageal lymph node was found by F-18 FDG PET/CT, and the nuclear medicine physician interpreted the paraesophageal lymph node as lymphoma involvement. The SUV_max_ of the paraesophageal lymph was 4.53. The hypermetabolic paraesophageal lymph node was not histopathologically confirmed, but the physician clinically upstaged the patient for effective treatment. The patient was HP-negative and was treated with radiation therapy and chemotherapy.

**Figure 1:**
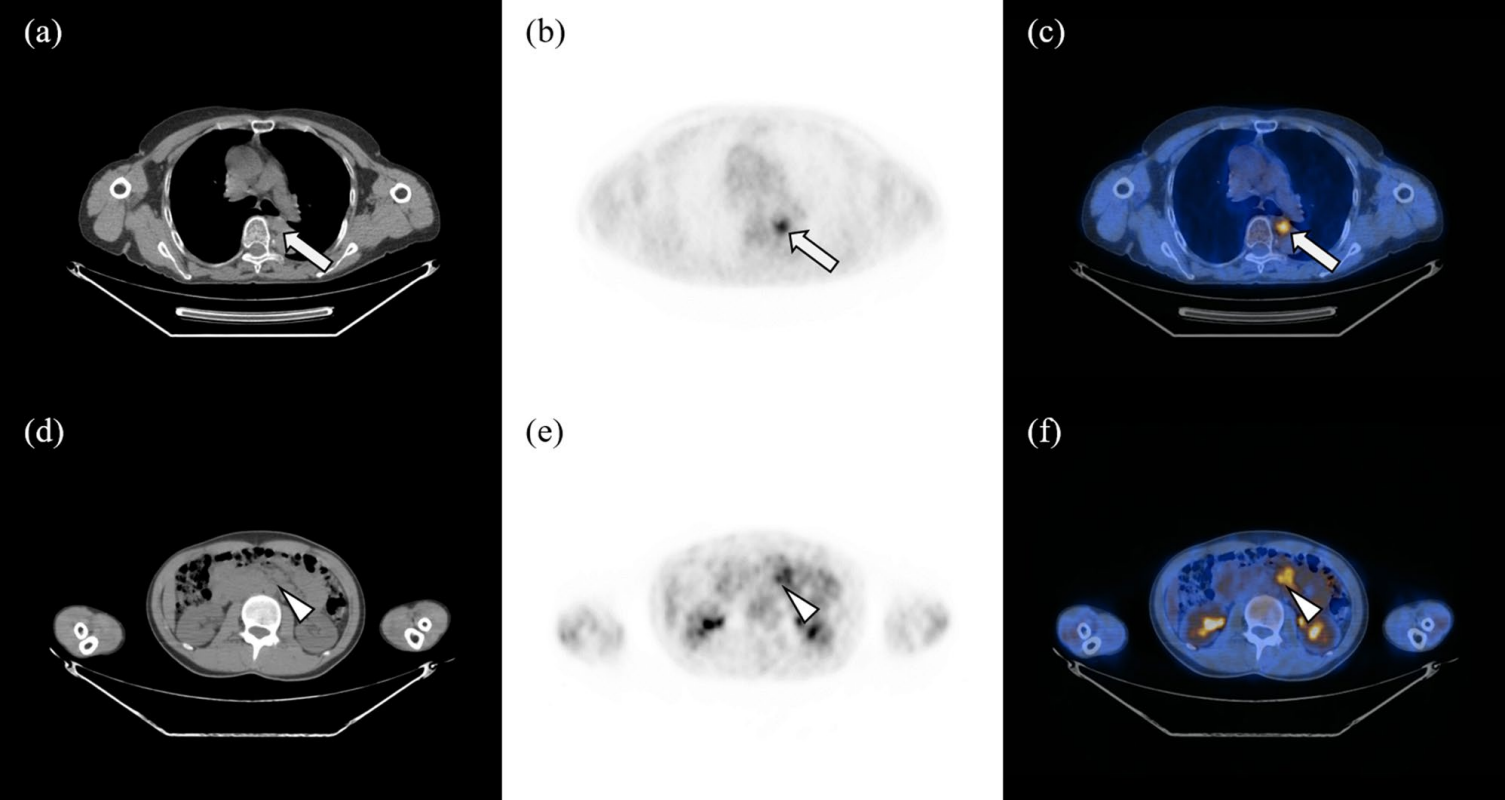
Non-contrast CT, F-18 FDG PET, and fusion images of two patients with correct upstaging by F-18 FDG PET/CT. (**a**–**c**) First, a 71-year-old female patient diagnosed with gastric MALT lymphoma was classified as modified Ann Arbor stage IE with the exception of F-18 FDG PET/CT during initial workup. F-18 FDG PET/CT showed a hypermetabolic paraesophageal lymph node consistent with lymphoma involvement (arrow, SUV_max_ 4.53). The hypermetabolic paraesophageal lymph node was not histopathologically confirmed, and the patient was clinically upstaged from clinical stage IE to IIIE. (**d**–**f**) Second, a 23-year-old female patient diagnosed with gastric MALT lymphoma was classified as modified Ann Arbor stage IE with the exception of F-18 FDG PET/CT during initial workup. F-18 FDG PET/CT showed a hypermetabolic mesenteric lymph node with high possibility of lymphoma involvement (arrow head, SUV_max_ 3.98). The hypermetabolic mesenteric lymph node was not histopathologically confirmed, but the clinical stage of the patient was changed from IE to IIE by F-18 FDG PET/CT. This figure was made using Advantage Workstation VolumeShare 7 (AW 4.7, GE Healthcare; https://www.gehealthcare.com/products/advanced-visualization/platforms/aw-volumeshare-7). CT, computed tomography; F-18 FDG, fluorine-18-fluorodeoxyglucose; PET, positron emission tomography; MALT, mucosa-associated lymphoid tissue; SUV_max_, maximum standardized uptake value.

The second case was detection of lymphoma involvement on pretherapeutic F-18 FDG PET/CT for a lesion that was not found in the clinical staging workup excluding F-18 FDG PET/CT. A 23-year-old female patient diagnosed with gastric MALT lymphoma was classified as modified Ann Arbor stage IE with the exception of F-18 FDG PET/CT during initial staging workup. A hypermetabolic mesenteric lymph node was found on F-18 FDG PET/CT, and the SUV_max_ of the mesenteric lymph node was 3.98. The mesenteric lymph node was not histopathologically confirmed, but it was clinically considered lymphoma involvement. Therefore, the modified Ann Arbor stage of the patient was changed from IE to IIE by F-18 FDG PET/CT. The F-18 FDG PET/CT images of the two patients are shown in Fig. 1.

### Predictive values of PET parameters and clinical parameters

**Figure 2:**
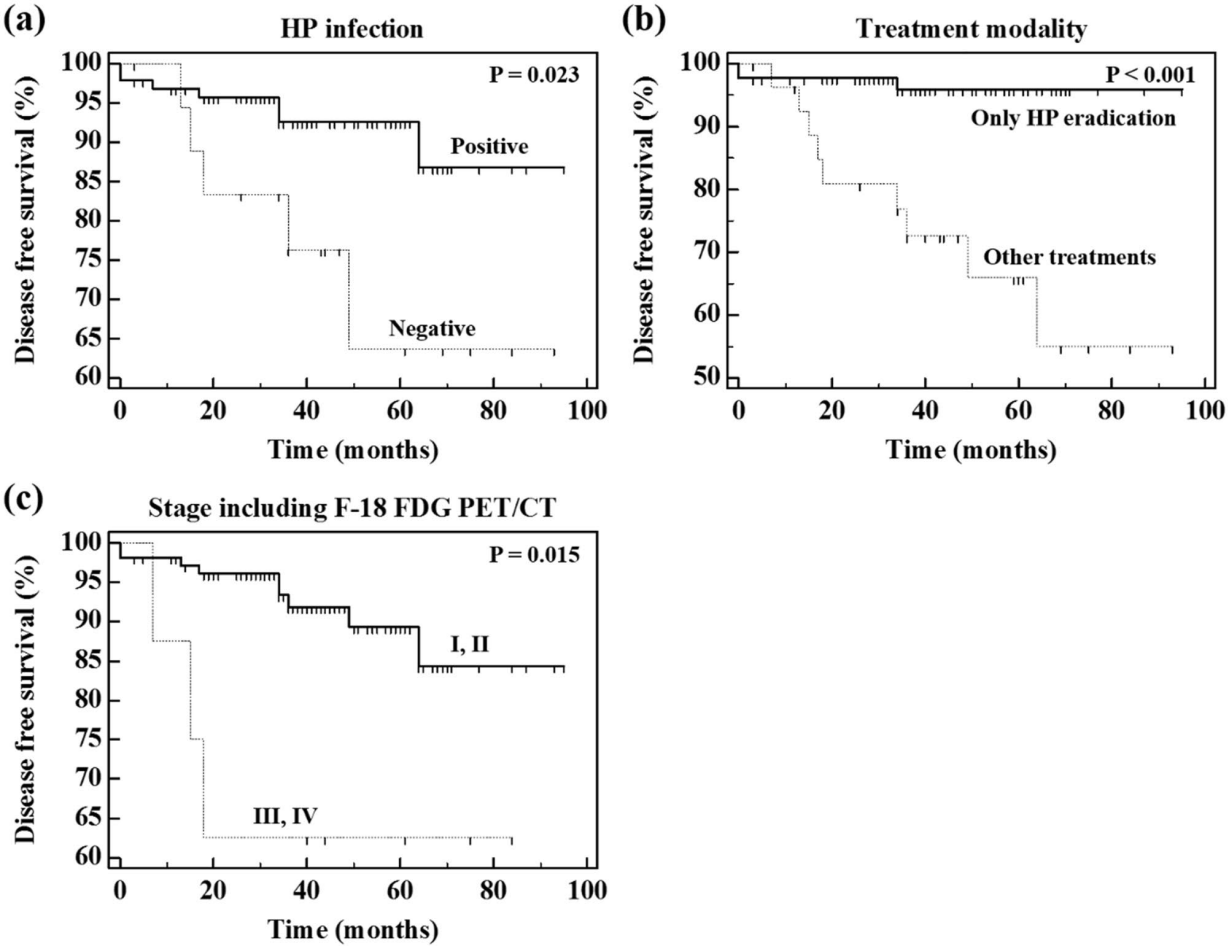
Kaplan–Meier curves of DFS with respect to HP infection (**a**), treatment modality (**b**), and stage including F-18 FDG PET/CT (**c**). This figure was made using MedCalc statistical software version 12.7.5.0 for Windows (Ostend, Belgium; https://www.medcalc.org). DFS, disease free survival; HP, *helicobacter pylori*; F-18 FDG, fluorine-18-fluorodeoxyglucose; PET, positron emission tomography; CT, computed tomography.

**Figure 3:**
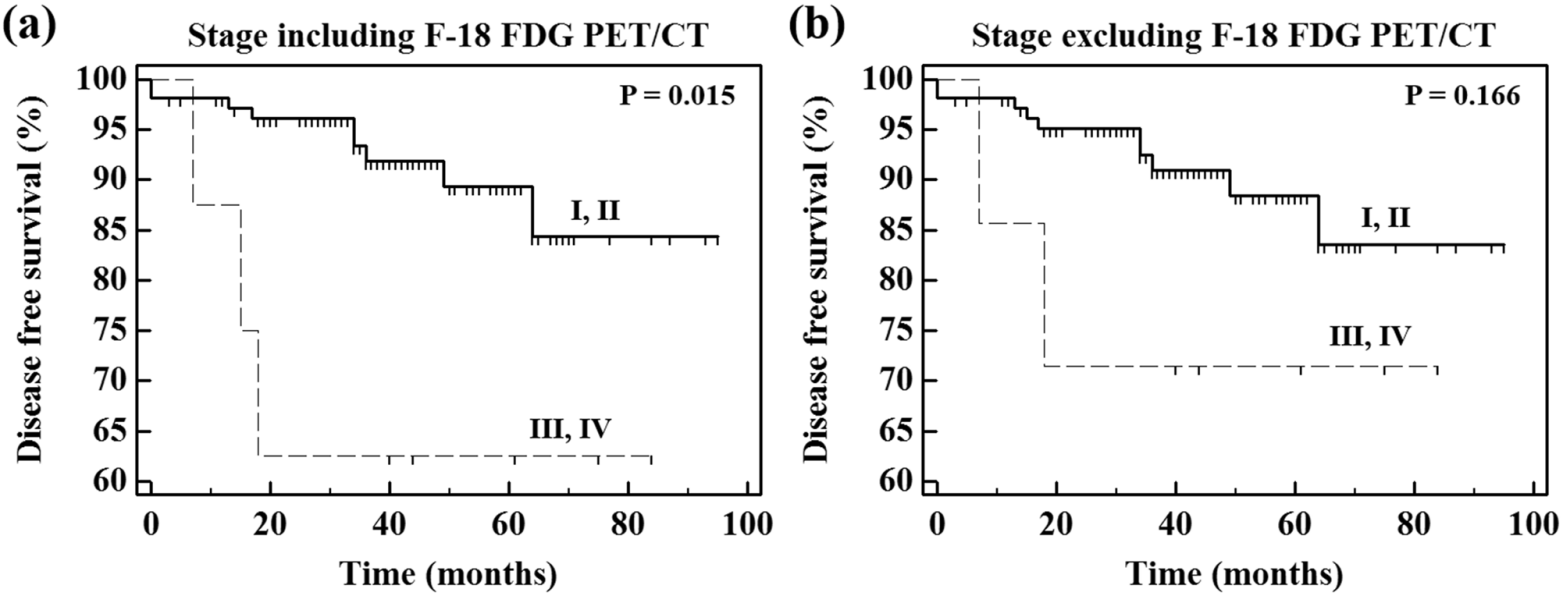
Comparison of Kaplan–Meier curves with respect to stage including F-18 FDG PET/CT (**a**) and stage excluding F-18 FDG PET/CT (**b**). This figure was made using MedCalc statistical software version 12.7.5.0 for Windows (Ostend, Belgium; https://www.medcalc.org). F-18 FDG, fluorine-18-fluorodeoxyglucose; PET, positron emission tomography; CT, computed tomography.

Univariate analysis for DFS using the Kaplan–Meier method was performed for all 16 parameters including 13 clinical parameters and three PET parameters. The optimal cutoff values yielding maximal sensitivity plus specificity for SUV_max_ and T/N ratio of gastric MALT lymphoma were calculated by time-dependent ROC curve analysis, and the optimal cutoff value for SUV_max_ of gastric MALT lymphoma was 6.31, and the optimal cutoff value for T/N ratio of gastric MALT lymphoma was 2.36. Of the 16 parameters, we found three parameters with P values less than 0.05 by univariate analysis. These three parameters represented statistically significant differences and included the following: HP infection (P = 0.023; 95% confidence interval = 0.771–15.591; hazard ratio = 3.467), treatment modality (P < 0.001; 95% confidence interval = 2.416–32.704; hazard ratio = 8.889), and stage including F-18 FDG PET/CT (P = 0.015; 95% confidence interval = 0.482–39.779; hazard ratio = 4.378). Kaplan–Meier curves for these three parameters are shown in Fig. 2. We identified better prognosis for DFS in HP-positive, only HP eradication, and stage I, II including F-18 FDG PET/CT. Statistically significant differences were not identified between the three PET parameters. Although there were no independent prognostic factors for DFS among the PET parameters, we found that stage including F-18 FDG PET/CT (P = 0.015) was more effective for predicting the DFS than stage excluding F-18 FDG PET/CT (P = 0.166) in the univariate analysis. The results of the univariate analysis are summarized in Table 2, and comparison of Kaplan–Meier curves with respect to stage including F-18 FDG PET/CT and stage excluding F-18 FDG PET/CT are presented in Fig. 3.

**Table 2:** Univariate analysis for DFS.

Parameter	HR (95% CI)	P value
Age (< 60 years vs. ≥ 60 years)		0.885
Sex (men vs. women)		0.837
β-2 microglobulin (normal vs. elevation)		0.330
Hemoglobin (normal vs. anemia)		0.897
LDH (normal vs. elevation)		0.227
Bone marrow involvement (positive vs. Negative)		0.407
B symptom (positive vs. negative)		0.392
IPI score (2 vs. 0.1)		0.270
Extragastric spread (positive vs. negative)		0.118
HP infection (negative vs. positive)	3.467 (0.771–15.591)	0.023*
Treatment modality (other treatments vs. only HP eradication)	8.889 (2.416–32.704)	< 0.001*
Stage including F-18 FDG PET/CT (III, IV vs. I, II)	4.378 (0.482–39.779)	0.015*
Stage excluding F-18 FDG PET/CT (III, IV vs. I, II)		0.166
SUV_max_ of gastric MALT lymphoma (≥ 6.31 vs. < 6.31)		0.255
T/N ratio of gastric MALT lymphoma (≥ 2.36 vs. < 2.36)		0.494
F-18 FDG uptake pattern of gastric lesion (Diffuse vs. Focal)		0.498

DFS, disease free survival; HR, Hazard ratio; CI, confidence interval; LDH, lactate dehydrogenase; IPI, international prognostic index; HP, *helicobacter pylori*; F-18 FDG, fluorine-18-fluorodeoxyglucose; PET, positron emission tomography; CT, computed tomography; SUV_max_, maximum standardized uptake value; MALT, mucosa-associated lymphoid tissue; T/N ratio, tumor-to-normal tissue ratio.

*Univariate analysis using Kaplan–Meier method with a log-rank test, P < 0.05.

Multivariate analysis using Cox proportional-hazards regression was performed with three parameters that showed statistically significant differences in the univariate analysis. Of the three parameters, only treatment modality showed a significant difference (P = 0.003; 95% confidence interval = 2.205–49.893; hazard ratio = 10.488). Therefore, treatment modality was an independent prognostic factor for predicting DFS in patients with gastric MALT lymphoma without HT. On the other hand, PET parameters were identified to be not independent prognostic factors for DFS. The results of multivariate analysis are summarized in Table 3.

**Table 3:** Multivariate analysis for DFS.

Parameter	HR (95% CI)	P value
HP infection (negative vs. positive)	0.677 (0.184–2.493)	0.559
Treatment modality (other treatments vs. only HP eradication)	10.488 (2.205–49.893)	0.003*
Stage including F-18 FDG PET/CT (III, IV vs. I, II)	1.274 (0.320–5.073)	0.732

DFS, disease free survival; HR, hazard ratio; CI, confidence interval; HP, *helicobacter pylori*; F-18 FDG, fluorine-18-fluorodeoxyglucose; PET, positron emission tomography; CT, computed tomography.

*Multivariate analysis using Cox proportional-hazards regression with enter method, P < 0.05.

### Subgroup analysis for prognostic values between two different PET/CT scanners

Because two different PET/CT scanners (Discovery LS and Discovery STE, GE Healthcare, Milwaukee, WI, USA) were used in the present study, subgroup analysis for prognostic values was performed for each PET/CT scanner. We found that patient characteristics did not significantly differ between the two different PET/CT scanners. Treatment modality was identified as a prognostic factor for two different PET/CT scanners. Significant differences were identified in the univariate analysis of stage including F-18 FDG PET/CT and HP infection on Discovery STE, but no significant differences were identified in the univariate analysis of stage including F-18 FDG PET/CT and HP infection on Discovery LS. Because only 34 patients underwent Discover LS PET/CT scanner, and there were only two recurrent cases. This small sample size resulted in low statistical power. Patient characteristics and results of univariate and multivariate analysis in patients who were examined with two different PET/CT scanner are summarized in Supplementary Tables S1 and S2.

## Discussion

F-18 FDG PET/CT is a non-invasive imaging modality that is used for staging, restaging, and treatment response evaluation for various cancers^5^. It has also played important roles in diagnosis, staging, restaging, prognostic evaluation, bone marrow involvement, and treatment decisions in patients with lymphoma^26–31^. The roles of F-18 FDG PET/CT in staging, treatment evaluation, and aggressive transformation of indolent lymphoma in aggressive lymphoma are well known. On the other hand, there is a lack of evidence for the usefulness of F-18 FDG PET/CT in indolent lymphoma, as it is associated with high false positive rates and cost^32^. The clinical role of F-18 FDG PET/CT in MALT lymphoma, a type of indolent lymphoma, is still controversial^5^. Because a high percentage of gastric MALT lymphoma was not accompanied by HT, study of the clinical role of F-18 FDG PET/CT in gastric MALT lymphoma without HT is needed. This is the first study to investigate the role in staging of pretherapeutic F-18 FDG PET/CT in patients with gastric MALT lymphoma without HT. In the present study, two patients (1.7%) were upstaged by pretherapeutic F-18 FDG PET/CT. Although only two patients were upstaged by F-18 FDG PET/CT, this technique was useful to identify patients who needed to be upstaged and increase the opportunity for these patients to receive effective treatment. This was also the first study to investigate the prognostic value of pretherapeutic F-18 FDG PET/CT for DFS in patients with gastric MALT lymphoma without HT. We identified no prognostic factors among the PET parameters, while only treatment modality was identified as a significant prognostic factor for DFS.

When measuring SUV_mean_s of normal gastric background in patients who underwent Discovery STE and Discovery LS PET/CT scanners, the mean value of SUV_mean_s of normal gastric background in patients who underwent Discovery LS PET/CT scanner were slightly higher than that in patients who underwent Discovery STE PET/CT scanner. However, no significant statistical differences were observed in the mean values of SUV_mean_s measured between the two different PET/CT scanners (P = 0.075). Comparison of SUV_mean_s of normal gastric background between all patients, patients who underwent Discovery STE, and patients who underwent Discovery LS are presented in Supplementary Figure S1. The T/N ratio of gastric MALT lymphoma was used to reduce the heterogeneity between patients and PET/CT scanners. The target-to-background SUV such as T/N ratio is independent from administered radioactivity and body weight of patient, and it has the advantage to overwhelm some limitations of absolute SUV values^33–36^. However, as shown in SUV_max_ of gastric MALT lymphoma (P = 0.255) in the univariate analysis, T/N ratio of gastric MALT lymphoma (P = 0.494) was also not identified as a significant prognostic factor in the univariate analysis. Comparison of SUV_max_s and T/N ratios of gastric MALT lymphoma between all patients, patients who underwent Discovery STE, and patients who underwent Discovery LS are presented in Supplementary Figure S2. However, F-18 FDG PET/CT was considered to play a prognostic role in this study, because stage including F-18 FDG PET/CT (P = 0.015) better reflected prognosis than stage excluding F-18 FDG PET/CT (P = 0.166) in univariate analysis. When the stage was divided into I-II and III-IV, only one was upstaged from stage I-II to III-IV by F-18 FDG PET/CT. The one upstaged patient had relapsed, and DFS of the patient was only 15 months. As the one patient with poor prognosis was upstaged from stage I, II to stage III, IV, the prognosis for stage I, II appeared to be better and the prognosis for stage III, IV appeared to be worse. Furthermore, the prognosis for stage III, IV including F-18 FDG PET/CT was significantly lower than the prognosis for stage III, IV excluding F-18 FDG PET/CT in Fig. 3. Since the number of patients with stage III, IV was less than 10% of all patients, the change in prognosis was considered to be significant according to whether F-18 FDG PET/CT was included or not. Therefore, one patient with poor prognosis was upstaged from stage I, II to stage III, IV, the prognosis of a small number of stage III, IV patients became more acutely worse.

In a previous study of 42 patients with primary gastric lymphoma including 32 with gastric DLBCL and 10 with gastric MALT lymphoma, nine (21.4%) were upstaged by F-18 FDG PET/CT^20^. Of the nine upstaged patients, seven were diagnosed with gastric DLBCL and two with gastric MALT lymphoma. The upstaging rate in gastric MALT lymphoma (20.0%) using F-18 FDG PET/CT was slightly lower than that in gastric DLBCL (21.9%). In a previous study of 69 patients with gastric MALT lymphoma, five (14%) were upstaged by F-18 FDG PET/CT^4^. Three of them were initially diagnosed with stage I and were upstaged to stage II or III by F-18 FDG PET/CT results identifying nodal involvement. Another patient was upstaged from stage I to stage IV after the confirmation of a pathological colon F-18 FDG uptake proven to be MALT lymphoma after biopsy. Another patient was upstaged from stage III to stage IV as a result of lungs involvement recognized with F-18 FDG PET/CT and then pathologically confirmed by biopsy. In another previous study of 142 patients with gastric MALT lymphoma, there was one case (0.7%) in which additional lymphoma involvement of the cervical lymph nodes was identified by F-18 FDG PET/CT, and the patient was upstaged from Ann Arbor stage I to III^14^. In the present study of patients with gastric MALT lymphoma without HT, only two (1.7%) were upstaged by F-18 FDG PET/CT. The one identified an additional lymphoma involvement in paraesophageal lymph node and the other one identified an additional lymphoma involvement in mesenteric lymph node by F-18 FDG PET/CT. The additional involvement sites were not identified by conventional CT scans, and it was not possible to find upstaged patients without F-18 FDG PET/CT. Accurate staging in patients with gastric MALT lymphoma without HT using F-18 FDG PET/CT was critical for effective treatments. Although the proportion of upstaging patients with gastric MALT lymphoma without HT by F-18 FDG PET/CT was low, the treatments of upstaged patients were changed by F-18 FDG PET/CT. In case of HP-positive and modified Ann Arbor stage IE, only HP eradication was performed. However, patients whose stage was raised from stage I to stage II-IV by F-18 FDG PET/CT received additional treatments such as radiation therapy, chemotherapy, and surgery. Therefore, stage including F-18 FDG PET/CT is important in patient with gastric MALT lymphoma without HT. In a previous study of 86 patients with primary gastric lymphoma including 34 with gastric MALT lymphoma and 52 with gastric DLBCL, the SUV_max_ of primary gastric lymphoma graded higher than 5.2 was confirmed to suggest poor prognosis^6^. In a previous study of 18 patients with HP infected low-grade gastric MALT lymphoma, the baseline SUV of two patients with failed HP eradication was significantly higher than that of patients with successful HP eradication, and changes in SUV of patients with complete response after treatment were significantly greater than in patients who failed the treatment^7^. In a previous study of 16 patients with gastric MALT lymphoma, SUV_max_ and proportion of focal F-18 FDG uptake of gastric MALT lymphoma were higher than those of the control group, and proportion of diffuse F-18 FDG uptake was lower than in the control group^37^. Depressed tumors and protruding tumors were mostly observed to have focal F-18 FDG uptake, and chronic gastritis-like tumors were mostly observed to have diffuse F-18 FDG uptake^37^. In this study, PET parameters including SUV_max_ of gastric MALT lymphoma, T/N ratio of gastric MALT lymphoma, and F-18 FDG uptake pattern of gastric lesion did not predict DFS.

**Figure 4:**
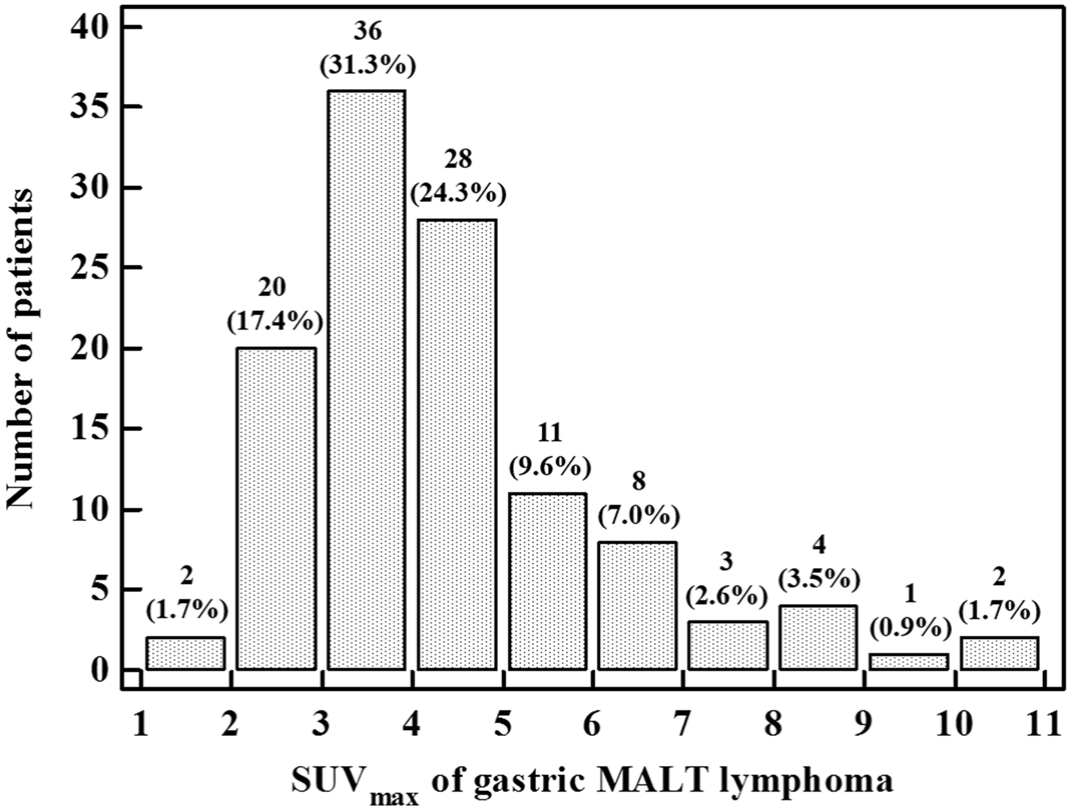
SUV_max_ distribution histograms of gastric MALT lymphoma. This figure was made using MedCalc statistical software version 12.7.5.0 for Windows (Ostend, Belgium; https://www.medcalc.org). SUV_max_, maximum standardized uptake value; MALT, mucosa-associated lymphoid tissue.

Gastric MALT lymphoma without HT has varying but generally low FDG avidity due to tumor size, depth of tumor invasion, morphologic features, and histologic features^4,5,14^, and background F-18 FDG uptake of the stomach can mask the F-18 FDG avidity of gastric MALT lymphoma^4,38–40^. Therefore, it is difficult to analyze the SUV_max_ and the T/N ratio of gastric MALT lymphoma. Small tumors emit weak metabolic signals due to the partial volume effect, and thus superficial and small lesions can yield false positive results^5,14^. Depth of tumor invasion has an important association with the proportion of F-18 FDG avid lesions, and F-18 FDG avidity increases as tumor invasion deepens^14^. Among morphologic features, the F-18 FDG avidity of protrusion, polyps, and mass-forming lesions was higher than that of superficial or chronic gastritis-like lesions, and different morphologic features show varying F-18 FDG avidity^4,5^. Significant associations also exist between F-18 FDG avidity and histologic features such as Ki-67 in gastric MALT lymphoma^4^. Furthermore, the F-18 FDG avidity of gastric MALT lymphoma is associated with plasmacytic differentiation, clinical stage classification, IPI, and MALT-IPI^5^. Background F-18 FDG uptakes of the stomach including physiologic uptake, uptakes related to infection and inflammation, and F-18 FDG uptake associated with pathogenesis can mask the F-18 FDG avidity of gastric MALT lymphoma^4,38–40^. The water gastric distention method, which involves drinking water before F-18 FDG PET/CT, can reduce the physiologic uptake of the stomach^41^, while delayed-time-point F-18 FDG PET/CT improves the detectability of MALT lymphoma when used instead of standard-time-point F-18 FDG PET/CT^42^. In a previous study, the mean SUV_max_s of stomach ranged from 2.71 to 4.36, depending on whether chronic gastritis and HP infection were present^38^. In this study, there were 86 (74.7%) patients with SUV_max_ less than 5, and the SUV_max_ of gastric MALT lymphoma overlapped with the SUV_max_ associated with the background uptake of the stomach. The SUV_max_ distribution histogram of gastric MALT lymphoma is shown in Fig. 4. Analyses using another PET parameter, F-18 FDG uptake pattern of gastric lesions, have limitations. In a previous study, 81% of gastric MALT lymphomas were superficial lesions, and 76% of non-gastric MALT lymphomas were mass-forming lesions^14^. Because F-18 FDG avid lesions were observed in mass-forming rather than superficial lesions, the presence of mass-forming lesions was important for determining the proportion of F-18 FDG avid lesions^14^. As gastric MALT lymphoma includes high proportions of superficial lesions, the F-18 FDG avidity of gastric MALT lymphoma is lower than that of other organs^14^. Superficial lesions and the low F-18 FDG avidity of gastric MALT lymphoma make it difficult to use the uptake pattern of gastric lesion to determine prognosis. In this study, the proportion of diffuse uptake of gastric lesions (58.3%) was higher than the proportion of focal uptake of gastric lesions (41.7%). Therefore, our results suggest that SUV_max_ of gastric MALT lymphoma, T/N ratio of gastric MALT lymphoma, and F-18 FDG uptake pattern of gastric lesion are not helpful for predicting prognosis in patients with gastric MALT lymphoma without HT.

In the present study, treatment modality was identified as an independent prognostic factor for DFS in patients with gastric MALT lymphoma without HT, and only HP eradication was better than other treatments regarding prognosis. The pathogenesis of gastric MALT lymphoma is known in terms of infectious background, autoimmune disease, and genetic abnormalities^43^. Some lymphomas are associated with microbial infections, and the most representative of these is HP infection-related gastric MALT lymphoma^44^. HP infection was found in almost 90% of gastric MALT lymphoma, and chronic HP infection can cause subsequent chronic atrophic gastritis, gastric ulcer, adenocarcinoma, and MALT lymphoma^43,45^. Generally, only HP eradication therapy is performed for HP-positive and modified Ann Arbor stage IE, and radiation therapy is performed if continuous HP eradication fails^22^. Among patients who had HP eradication, patients over 40 years of age or with duodenal ulcers had high HP eradication rate^46^. HP-negative gastric MALT lymphoma has also successfully been treated by HP eradication therapy^47^. t(11;18)(q21;q21), nuclear expression of BCL10 and NF-κB were valuable immunohistochemical biomarkers to predict first-line antibiotic response in patients with HP-negative low-grade gastric MALT lymphoma^48^. In cases of modified Ann Arbor stage II-IV or HP-negative gastric MALT lymphoma, additional treatments such as radiation therapy, chemotherapy, and surgery are performed^22^. HP-positive status and low clinical stage are associated with better prognosis in univariate analysis, and only HP eradication was performed when HP-positive and Ann Arbor stage IE were present. Therefore, only HP eradication resulted in better prognosis than other treatments, and treatment modality was identified as an independent prognostic factor in patients with gastric MALT lymphoma without HT.

This study has some limitations. This was a single-center retrospective study, so there was potential for selection bias. This study included a small number of subjects, so there was potential for low statistical power. In addition, two different PET/CT scanners were used in the present study, statistical differences between two different PET/CT scanners were identified. SUVs are also affected by other factors, which could explain any differences in SUVs for the two different PET/CT scanners. These limitations can be overcome by large scale or multi-center prospective studies and SUV harmonization in the future.

In conclusion, this is the first study to investigate the role in staging and prognostic value of pretherapeutic F-18 FDG PET/CT in patients with gastric MALT lymphoma without HT. In the present study, pretherapeutic F-18 FDG PET/CT played an important role in enabling upstaging of two patients, and treatments of the upstaged patients were changed according to the stage. However, the PET parameters of gastric MALT lymphoma lesions were not associated with survival. There were limitations in using PET parameters due to variation, but we observed generally low F-18 FDG avidity of gastric MALT lymphoma associated with multiple causes, background F-18 FDG uptake of stomach, and superficial lesion of gastric MALT lymphoma. The stage including F-18 FDG PET/CT findings was a better prognostic factor than the stage excluding F-18 FDG PET/CT, which suggests that F-18 FDG PET/CT may have a prognostic role in gastric MALT lymphoma without HT.

## Supplementary Information

Supplementary Information 1.

Supplementary Information 2.

Supplementary Information 3.

## Data Availability

The datasets used and/or analyzed during the current study are available from the corresponding author on reasonable request.
