# Supplementary material for: Role in staging and prognostic value of pretherapeutic F-18 FDG PET/CT in patients with gastric MALT lymphoma without high-grade transformation
Source: Sci Rep. 2021 Apr 29;11:9243. doi: 10.1038/s41598-021-88815-2 (PMC8084924; doi:10.1038/s41598-021-88815-2)
Supplement: Supplementary file 1 — Supplementary Information 1. [file 41598_2021_88815_MOESM1_ESM.docx]

<Original Research Article>

**Role in staging and prognostic value of pretherapeutic F-18 FDG PET/CT in patients with gastric MALT lymphoma without high-grade transformation**

Yong-Jin Park^1^, Seung Hyup Hyun^1^, Seung Hwan Moon^1^, Kyung-Han Lee^1^, Byung Hoon Min^2^, Jun Haeng Lee^2^, Won Seog Kim^3^, Seok Jin Kim^3^, and Joon Young Choi^1*^

^1^Department of Nuclear Medicine and Divisions of ^2^Gastroenterology and ^3^Hematology-Oncology, Department of Internal Medicine, Samsung Medical Center, Sungkyunkwan University School of Medicine, Seoul, Korea, 06351

Correspondence and requests for materials should be addressed to J.Y.C. (email: jynm.choi@samsung.com)

**First Author:** Yong-Jin Park, MD

Department of Nuclear Medicine, Samsung Medical Center, 81, Irwon-ro, Gangnam-gu, Seoul, Republic of Korea, 06351

**Telephone:** +82-2-3410-2627, **Fax:** + 82-2-3410-2639, **E-mail:** yongjin.park@hanmail.net

**Corresponding Author:** Joon Young Choi, MD, PhD

Department of Nuclear Medicine, Samsung Medical Center, Sungkyunkwan University School of Medicine, 81, Irwon-ro, Gangnam-gu, Seoul, Republic of Korea, 06351

**Telephone:** +82-2-3410-2648, **Fax:** + 82-2-3410-2639, **E-mail:** jynm.choi@samsung.com

**Supplementary Figure Legends**

**Supplementary Figure S1.** Comparison of SUV_mean_s of normal gastric background between all patients, patients who underwent Discovery STE, and patients who underwent Discovery LS. This figure was made using MedCalc statistical software version 12.7.5.0 for Windows (Ostend, Belgium; https://www.medcalc.org).

Abbreviations: SUV_mean_, mean standardized uptake value.

**Supplementary Figure S2.** Comparison of SUV_max_s (a) and T/N ratios (b) of gastric MALT lymphoma between all patients, patients who underwent Discovery STE, and patients who underwent Discovery LS. This figure was made using MedCalc statistical software version 12.7.5.0 for Windows (Ostend, Belgium; https://www.medcalc.org).

Abbreviations: SUV_max_, maximum standardized uptake value; T/N ratio, tumor-to-normal tissue ratio; MALT, mucosa-associated lymphoid tissue.

**Supplementary Table S1.** Baseline characteristics of patients according to the type of PET/CT scanner.

|  |  | Discovery STE (81) | | Discovery LS (34) | |
| --- | --- | --- | --- | --- | --- |
| Parameter |  | N (Proportion) | Mean ± SD (Range) | N (Proportion) | Mean ± SD (Range) |
| Age (years) |  |  | 52 ± 10 (28 – 75) |  | 54 ± 15 (23 – 77) |
| Sex | Men | 37 (45.7) |  | 16 (47.1) |  |
|  | Women | 44 (54.3) |  | 18 (52.9) |  |
| Recurrence |  | 10 (12.3) |  | 2 (5.9) |  |
| DFS (months) |  |  | 38 ± 20 (0 – 95) |  | 50 ± 20 (5 – 87) |
| β-2 microglobulin (mg/L) |  |  | 1.52 ± 0.63 (0.54 – 4.57) |  | 2.03 ± 1.39 (0.66 – 7.37) |
| Hemoglobin (g/dL) |  |  | 13.6 ± 1.8 (6.5 – 17.7) |  | 13.4 ± 2.4 (7.7 – 17.3) |
| LDH (IU/L) |  |  | 352 ± 78 (232 – 702) |  | 371 ± 77 (262 – 560) |
| Bone marrow involvement |  | 3 (3.7) |  | 1 (2.9) |  |
| B symptom |  | 3 (3.7) |  | 2 (5.9) |  |
| IPI score | 0 | 53 (65.4) |  | 19 (55.9) |  |
|  | 1 | 22 (27.2) |  | 13 (38.2) |  |
|  | 2 | 6 (7.4) |  | 2 (5.9) |  |
| Extragastric spread |  | 12 (14.8) |  | 3 (8.8) |  |
| HP infection |  | 67 (82.7) |  | 29 (85.3) |  |
| Treatment modality | Only HP eradication | 61 (75.3) |  | 26 (76.5) |  |
|  | Other treatments | 20 (24.7) |  | 8 (23.5) |  |
| Stage including F-18 FDG PET/CT | I | 72 (88.9) |  | 31 (91.2) |  |
|  | II | 2 (2.5) |  | 2 (5.9) |  |
|  | III | 1 (1.2) |  | 0 (0) |  |
|  | IV | 6 (7.4) |  | 1 (2.9) |  |
| Stage excluding F-18 FDG PET/CT | I | 73 (90.1) |  | 32 (94.1) |  |
|  | II | 2 (2.5) |  | 1 (2.9) |  |
|  | III | 0 (0) |  | 0 (0) |  |
|  | IV | 6 (7.4) |  | 1 (2.9) |  |
| SUV_max_ of gastric MALT lymphoma |  |  | 4.32 ± 1.75 (1.45 – 10.24) |  | 4.45 ± 1.81 (2.00 – 8.91) |
| T/N ratio of gastric MALT lymphoma |  |  | 2.03 ± 0.85  (0.89 – 6.51) |  | 1.93 ± 0.67  (0.95 – 4.16) |
| F-18 FDG uptake pattern of gastric lesion | Diffuse | 49 (60.5) |  | 18 (52.9) |  |
|  | Focal | 32 (39.5) |  | 16 (47.1) |  |

Abbreviations: SD, standard deviation; DFS, disease free survival; LDH, lactate dehydrogenase; IPI, international prognostic index; HP, *helicobacter pylori*; F-18 FDG, fluorine-18-fluorodeoxyglucose; PET, positron emission tomography; CT, computed tomography; SUV_max_, maximum standardized uptake value; MALT, mucosa-associated lymphoid tissue; T/N ratio, tumor-to-normal tissue ratio.**Supplementary Table S2.** Univariate analysis and multivariate analysis in patients according to the type of PET/CT scanner.

| Discovery STE (81) | Univariate analysis | | Multivariate analysis | |
| --- | --- | --- | --- | --- |
| Parameter | HR (95% CI) | P value | HR (95% CI) | P value |
| Age (< 60 years vs. ≥ 60 years) |  | 0.303 |  |  |
| Sex (Men vs. Women) |  | 0.778 |  |  |
| β-2 microglobulin (Normal vs. Elevation) |  | 0.994 |  |  |
| Hemoglobin (Normal vs. Anemia) |  | 0.957 |  |  |
| LDH (Normal vs. Elevation) |  | 0.294 |  |  |
| Bone marrow involvement (Positive vs. Negative) |  | 0.437 |  |  |
| B symptom (Positive vs. Negative) |  | 0.494 |  |  |
| IPI score (2 vs. 0.1) |  | 0.201 |  |  |
| Extragastric spread (Positive vs. Negative) |  | 0.113 |  |  |
| HP infection (Negative vs. Positive) | 3.356 (0.634 – 17.774) | 0.045* | 0.845 (0.186 – 3.847) | 0.828 |
| Treatment modality (Other treatments vs. Only HP eradication) | 6.999 (1.673 – 29.286) | 0.001* | 6.837 (1.166 – 40.076) | 0.034* |
| Stage including F-18 FDG PET/CT (III, IV vs. I, II) | 4.562 (0.499 – 41.680) | 0.015* | 1.394 (0.308 – 6.309) | 0.668 |
| Stage excluding F-18 FDG PET/CT (III, IV vs. I, II) |  | 0.156 |  |  |
| SUV_max_ of gastric MALT lymphoma (≥ 6.31 vs. < 6.31) |  | 0.343 |  |  |
| T/N ratio of gastric MALT lymphoma (≥ 2.36 vs. < 2.36) |  | 0.635 |  |  |
| F-18 FDG uptake pattern of gastric lesion (Diffuse vs. Focal) |  | 0.450 |  |  |
| Discovery LS (34) | Univariate analysis | |  | |
| Parameter | HR (95% CI) | P value |  |  |
| Age (< 60 years vs. ≥ 60 years) |  | 0.202 |  |  |
| Sex (Men vs. Women) |  | 0.919 |  |  |
| β-2 microglobulin (Normal vs. Elevation) |  | 1 |  |  |
| Hemoglobin (Normal vs. Anemia) |  | 0.689 |  |  |
| LDH (Normal vs. Elevation) |  | 0.589 |  |  |
| Bone marrow involvement (Positive vs. Negative) |  | 0.761 |  |  |
| B symptom (Positive vs. Negative) |  | 0.676 |  |  |
| IPI score (2 vs. 0.1) |  | 0.676 |  |  |
| Extragastric spread (Positive vs. Negative) |  | 0.676 |  |  |
| HP infection (Negative vs. Positive) |  | 0.228 |  |  |
| Treatment modality (Other treatments vs. Only HP eradication) | NA | 0.021* |  |  |
| Stage including F-18 FDG PET/CT (III, IV vs. I, II) |  | 0.773 |  |  |
| Stage excluding F-18 FDG PET/CT (III, IV vs. I, II) |  | 0.773 |  |  |
| SUV_max_ of gastric MALT lymphoma (≥ 6.31 vs. < 6.31) |  | 0.575 |  |  |
| T/N ratio of gastric MALT lymphoma (≥ 2.36 vs. < 2.36) |  | 0.549 |  |  |
| F-18 FDG uptake pattern of gastric lesion (Diffuse vs. Focal) |  | 0.828 |  |  |

Abbreviations: DFS, disease free survival; HR, Hazard ratio; CI, confidence interval; LDH, lactate dehydrogenase; IPI, international prognostic index; HP, *helicobacter pylori*; F-18 FDG, fluorine-18-fluorodeoxyglucose; PET, positron emission tomography; CT, computed tomography; SUV_max_, maximum standardized uptake value; MALT, mucosa-associated lymphoid tissue; T/N ratio, tumor-to-normal tissue ratio.

* P < 0.05 in univariate analysis and multivariate analysis.
